# Diffusion tensor imaging detects ventilation-induced brain injury in preterm lambs

**DOI:** 10.1371/journal.pone.0188737

**Published:** 2017-12-06

**Authors:** Dhafer M. Alahmari, Kyra Y. Y. Chan, Vanesa Stojanovska, Domenic LaRosa, Samantha K. Barton, Ilias Nitsos, Valerie Zahra, Jade Barbuto, Michael Farrell, Shigeo Yamaoka, James T. Pearson, Graeme R. Polglase

**Affiliations:** 1 Monash Biomedicine Discovery Institute and Department of Medical Imaging and Radiation Sciences, Monash University, Clayton, VIC, Australia; 2 Monash Biomedical Imaging, Monash University, Clayton, VIC, Australia; 3 Department of Diagnostic Imaging, King Saud Medical City, Riyadh, Saudi Arabia; 4 The Ritchie Centre, Hudson Institute of Medical Research and Department of Obstetrics and Gynaecology, Monash University, Clayton, VIC, Australia; 5 Department of Physiology, Monash University, Clayton, VIC, Australia; 6 Department of Cardiac Physiology, National Cerebral and Cardiovascular Center, Suita, Osaka, Japan; Cleveland Clinic, UNITED STATES

## Abstract

**Purpose:**

Injurious mechanical ventilation causes white matter (WM) injury in preterm infants through inflammatory and haemodynamic pathways. The relative contribution of each of these pathways is not known. We hypothesised that *in vivo* magnetic resonance imaging (MRI) can detect WM brain injury resulting from mechanical ventilation 24 h after preterm delivery. Further we hypothesised that the combination of inflammatory and haemodynamic pathways, induced by umbilical cord occlusion (UCO) increases brain injury at 24 h.

**Methods:**

Fetuses at 124±2 days gestation were exposed, instrumented and either ventilated for 15 min using a high tidal-volume (V_T_) injurious strategy with the umbilical cord intact (INJ; inflammatory pathway only), or occluded (INJ+UCO; inflammatory and haemodynamic pathway). The ventilation groups were compared to lambs that underwent surgery but were not ventilated (Sham), and lambs that did not undergo surgery (unoperated control; Cont). Fetuses were placed back *in utero* after the 15 min intervention and ewes recovered. Twenty-four hours later, lambs were delivered, placed on a protective ventilation strategy, and underwent MRI of the brain using structural, diffusion tensor imaging (DTI) and magnetic resonance spectroscopy (MRS) techniques.

**Results:**

Absolute MRS concentrations of creatine and choline were significantly decreased in INJ+UCO compared to Cont lambs (*P* = 0.03, *P* = 0.009, respectively); no significant differences were detected between the INJ or Sham groups and the Cont group. Axial diffusivities in the internal capsule and frontal WM were lower in INJ and INJ+UCO compared to Cont lambs (*P* = 0.05, *P* = 0.04, respectively). Lambs in the INJ and INJ+UCO groups had lower mean diffusivities in the frontal WM compared to Cont group (*P =* 0.04). DTI colour mapping revealed lower diffusivity in specific WM regions in the Sham, INJ, and INJ+UCO groups compared to the Cont group, but the differences did not reach significance. INJ+UCO lambs more likely to exhibit lower WM diffusivity than INJ lambs.

**Conclusions:**

Twenty-four hours after injurious ventilation, DTI and MRS showed increased brain injury in the injuriously ventilated lambs compared to controls. DTI colour mapping threshold approach provides evidence that the haemodynamic and inflammatory pathways have additive effects on the progression of brain injury compared to the inflammatory pathway alone.

## Introduction

Brain injury is a worldwide problem in preterm infants and is associated with a high risk of morbidity and mortality [1]. Children born very preterm have higher rates of cerebral palsy, educational disabilities and respiratory illnesses [2–4]. These lifelong morbidities cause substantial economic costs for individuals and countries [4,5]. Of 70% of surviving preterm infants, 58% have severe overall disability or mild disability with neuromotor abnormalities [6]. Despite significant improvements to neonatal care resulting in increased preterm survival, efforts to reduce or prevent neonatal morbidity and subsequent physical and neurodevelopmental disabilities have had little success so far.

The perinatal period is a critical time for heightened risk of preterm brain injury [7]. The transition from fetus to independent life in preterm infants is complicated by the immaturity of respiratory and cardiovascular systems, which can make the brain vulnerable to injury [8]. The requirement for respiratory support in the delivery room, which is extremely common in these infants [9], has been identified as a potential cause of preterm brain injury; termed ventilation-induced brain injury (VIBI) [10]. The risk of VIBI is amplified in infants who receive high tidal volumes (V_T_) in the delivery room, which occurs in up to 80% of infants in the delivery room receiving positive pressure ventilation [11]. Our studies have demonstrated that as little as 15 min of high V_T_ ventilation can increase early markers of brain injury through two key pathways: 1) initiation of a systemic inflammatory cascade resulting in cerebral inflammation and injury; and 2) haemodynamic instability resulting in fluctuations in cerebral blood flow and pressure [10,12]. Early markers of brain injury can be detected within 1–2 h after birth using histological techniques [12] as well as advanced magnetic resonance imaging (MRI) [13,14]. However, it is unlikely that infants can undergo MRI so soon after birth. It is not known whether the pathology observed within two hours of delivery manifests into gross brain injury 24 h later. Further, the relative contribution of the inflammatory or haemodynamic pathways of VIBI is not known.

To investigate how VIBI evolves over time we aimed to examine whether 15 min of high V_T_ ventilation results in significant microstructural brain injury 24 h later. Since VIBI occurs through two major pathways [12], we also aimed to determine the relative contribution of each pathway to brain injury. We hypothesised that structural brain injury evolves and is detectable by non-invasive MRI techniques on clinical 3T scanners at 24 h after high V_T_ ventilation induced to the fetal *in utero*. Further, we hypothesised that activation of the haemodynamic and inflammatory pathways would result in levels of WM injury that exceed levels of damage associated with the inflammatory pathway alone.

## Methods

### Ethics statement

The experimental protocol was approved by the Monash Medical Centre ‘A’ animal ethics committee at Monash University and conducted according to guidelines established by the National Health and Medical Research Council of Australia.

### Ventilation strategy, preterm delivery and stabilisation

Under sterile conditions, pregnant ewes at 124 ± 2 days of gestation (term ~ 148 days) were anaesthetised via intravenous injection of thiopentone sodium (20 mg/kg; Jurox, NSW, Australia), followed by tracheal intubation and delivery of inhalational anesthesia (isofluorane 1.5–2.5% in oxygenated air; Bomac Animal Health, NSW, Australia). Fetuses were exposed via laparotomy, and were instrumented for the placement of catheters into the left jugular vein and carotid artery as described previously [10,12]. The fetal chest was exteriorised and the fetus dried, intubated (4.0 mm cuffed endotracheal tube), lung liquid drained and randomised to one of three groups:

1. INJ: Lambs (*n* = 5) received 15 min of high V_T_ ventilation (targeting 12–15 mL/kg) initiating predominately the inflammatory pathway of VIBI.
2. INJ+UCO: Lambs (*n* = 7) received 15 min of high V_T_ ventilation (targeting 12–15 mL/kg) during which the umbilical cord was occluded. The removal of the placental circulation simulates umbilical cord clamping and triggers the cardiovascular transition at birth and its associated instability [15] resulting in initiation of the inflammatory and haemodynamic pathways of VIBI. At the end of the 15 minutes of injurious ventilation the occluder was removed and placental blood flow restored to the fetus.
3. Sham: The sham surgery group (*n* = 5) underwent catheterisation and intubation but did not receive mechanical ventilation.

A fourth group of lambs did not undergo the initial surgery or ventilation and were used as un-operated group (Cont; *n* = 6) to control for the surgical intervention.

Ventilation was conducted using a neonatal positive pressure ventilator (Babylog 8000+, Dräger, Lübeck, Germany) using heated, humidified air (Fisher and Paykel). Peak inflation pressure (PIP) was limited to 45 cmH_2_O with a positive end expiratory pressure (PEEP) of 5 cmH_2_O [15]. Ventilated lambs received a V_T_ of 7 mL/kg at 5 min, escalated to 10 mL/kg at 10 min and targeting 12–15 mL/kg (the PIP was limited to 45 cmH2O) by 15 min. Fetal blood-gas variables and oxygenation were monitored and maintained within normal newborn parameters. The ventilation was conducted under sterile conditions to prevent infection of the fetus.

After ventilation, the fetus was returned to the uterus and it was sutured closed. The jugular vein and carotid artery catheters were externalised through the ewe’s flank via a small incision [16] to allow periodic blood sampling. The abdominal and flank incisions were sutured closed and the ewe allowed to recover. Analgesia was provided to the ewe via buprenorphine (300ug/ml i.v., Reckitt Benckiser, UK) and a fentanyl patch (75 mg/h, Janssen-Cilag, NSW, Australia). At regular intervals over the proceeding 24 h, fetal arterial blood samples were collected to ensure fetal health by assessing the partial pressure of arterial carbon dioxide (PaCO_2_), oxygen (PaO_2_), oxygen saturation (SaO_2_), and pH (ABL30, Radiometer, Copenhagen, Denmark).

Twenty-four hours later, ewes were again anaesthetised as above, the fetus exteriorised via the same incision site, intubated, and ventilation initiated using volume guarantee set at 7 mL/kg and PEEP of 5 cmH_2_O. Lambs were ventilated for 3 minutes before the umbilical cord was clamped and surfactant (100 mg/kg, Curosurf^R^, Chiesi Pharma, Italy) was administered within the first 15 min of ventilation. The fraction of inspired oxygen was adjusted to maintain arterial oxygen saturation between 88–95%. The umbilical cord was cut 15 min after ventilation onset and the lambs transferred to the MRI. Ewes were euthanised immediately after the lamb was removed; lambs were euthanised after the MRI (sodium pentobarbitone 100 mg/kg i.v.).

### Magnetic resonance imaging

MRI acquisition protocols were conducted as per previously published [13,14]. Following stabilisation, lambs were transferred to the 3T MRI system (Siemens Skyra, Erlangen, Germany) at Monash Biomedical Imaging (Clayton, Australia), with a 15-channel radio frequency coil for transmission and reception. Lambs were placed in supine position, and ventilation was maintained using a BabyPAC portable and MR-compatible ventilator (Pneupac, Smiths Medical, UK). Brain MRI included structural imaging sequences (T1, T2), susceptibility-weighted imaging (SWI), DTI, and single-voxel MRS [13,14]. The total acquisition time was about 40 min.

We acquired images for anatomical localisation and identification of gross brain injury, including infarcts and haemorrhages. Whole-brain DTI was acquired using a single-shot, echo-planar imaging sequence with the following image parameters: repetition time (TR) = 11,400 ms; echo time (TE) = 99 ms; slice thickness = 1.2 mm; field of view = 154×154 mm; acquisition matrix = 128×128, giving a voxel size of 1.2×1.2×1.2 mm^3^, for five *b =* 0. Diffusion-encoding gradients were acquired in 30 directions with a *b* value of 1,000 s/mm^2^. The diffusion-weighted imaging was performed twice; five *b* = 0 volumes were acquired. A single-voxel spin-echo sequence was used to acquire MRS, localised on the supratentorial deep grey matter and central WM (TR = 2,000 ms, TE = 270 ms) with a voxel size of 15×15×20 mm^3^.

### MRI data analysis

All data were analysed using the FMRIB Software Library (FSL, FMRIB, Oxford, UK [17]). First, the FDT toolbox was used to correct all DTI data for eddy-current distortion, and then DTI parametric maps of fractional anisotropy (FA), axial diffusivity (AD), radial diffusivity (RD), and mean diffusivity (MD) were created with the FMRIB Diffusion Toolbox. All maps were co-registered with high-resolution T2 images of the brains using the Linear Image Registration Tool (FLIRT [18]), with the *b =* 0 image used as a reference [14].

ROIs were defined manually on the high-resolution T2 images of each lamb and then transformed to the corresponding FA, AD, MD, and RD maps to calculate the mean regional values for each lamb [13,14]. ROIs were placed in the thalamus (Th), periventricular white matter (PVWM), IC, frontal white matter (FWM), and cerebellum (CB) vermis, targeting the stalk and midline WM (S1 Fig). The mean value of the adjacent 4–5 slices that defined a given ROI volume was calculated for each ROI to test between-animal diffusivity variability. MATLAB R2013a (Mathworks, MA, USA) was then used to calculate the standard deviation (SD) for the ROIs of each region of the lamb brains to test within-animal variability (heterogeneity in DTI).

MRS data were processed with TARQUIN software to implement spectral fitting in the time domain [13,14]. Peak-area ratios for lactate (Lac) relative to NAA (total N-acetylaspartate and N-acetylaspartylglutamate), creatine (Cr, total phosphocreatine and free creatine), and choline (Cho, total glycerophosphocholine and phosphocholine) were calculated, along with major peak areas (Cr, NAA, Lac, and Cho).

DTI-ROI data showed more heterogeneity in the specified white matter regions of the lamb brains. To further examine this we utilised a colour-map threshold technique to determine where the low-diffusivity voxels were distributed in the DTI volumes, as previously described [14]. The total pixel intensity distribution for each brain was compared between the minimum and maximum intensities for 256 bins in all DTI images (S2 Fig) to produce the frequency distribution of MRI voxel intensity levels at each position [14]. Here, we applied a threshold to the mapped intensities in the overlay in all groups (width, 10%); the upper threshold (S2 Fig, green dashed line) was manually set at an intensity where voxels were almost completely absent across the brain in Cont lambs, but coloured voxels were still evident at the edges of the brain mask. The upper threshold was consistent with the 90^th^ percentile of intensities in the Cont lambs, with 3–5% overlap in observed pixel intensities among the groups (S2 Fig). This process permitted mapping and quantification of low-diffusivity voxels for AD, RD, and MD colour-map images.

### Statistical analyses

Physiological data were compared using a two-way repeated measure ANOVA and Holm-Sidak post-hoc analyses to determine significant interactions (Sigmaplot, Systat Software Inc). The mean and SEM of ROI voxel intensities were calculated, as well as one-way ANOVA was used to compare treatment groups with a significance level of *P*<0.05. Data are presented as means (box 5–95% CIs of mean) with maximum–minimum error bars. The SD was calculated for the ROIs in each brain to test within-animal variability and the heterogeneity of DTI diffusivities for differences between groups.

## Results

### Physiological parameters

**Fig 1:**
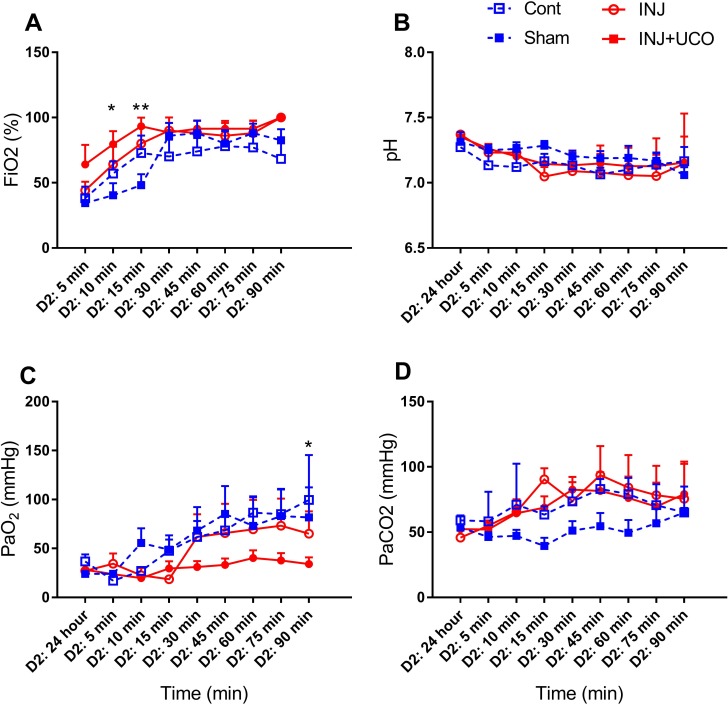
Physiological parameters. (A) Fraction of inspired oxygen (FiO_2_) in the unoperated control (Cont; blue open squares n = 6), sham surgery (Sham; blue closed squares n = 5), injurious ventilation (INJ; red open circles n = 5), and INJ + umbilical cord occlusion (INJ+UCO; red closed circles n = 7) groups. (B) pH. (C) Partial pressure of arterial oxygen (PaO_2_) and (D) carbon dioxide (PaCO_2_). *P<0.01, **P<0.001 vs INJ+UCO.

Fetal blood gases parameters at the time of intubation and cord clamping, body weight and sex were not different between groups (Table 1). Blood gases and haemodynamic data were recorded throughout the MRI examination. In brief, at 10 and 15 minutes after delivery INJ+UCO lambs required a higher fraction of inspired oxygen (FiO_2_) compared to the Cont group (*P* = 0.02 and *P* = 0.007, respectively; Fig 1A). However, pH, PaO_2_ and PaCO_2_ were not different between groups (*P* = 0.8, *P* = 0.1 and *P* = 0.2, respectively; Fig 1B–1D). Peak inflation pressure and positive end-expiratory pressure were not different between groups (*P* = 0.09 and *P* = 0.2, respectively). Alveolar-arterial differences in oxygen (AaDO_2_) were not different between groups at any time point (*P* = 0.1).

**Table 1:** Fetal characteristics at the time of intervention at 124 days of gestation.

Variable	Cont	Sham	INJ	INJ+UCO	*P* value
Sex (M/F)	5 / 1	3 / 2	4 / 1	1 / 6	-
Gestational age (d)	126 ± 1	126 ± 1	126 ± 1	126 ± 1	0.2
Body weight (kg)	3.4 ± 0.4	3.3 ± 0.2	3.6 ± 0.3	3.2 ± 0.4	0.3
SaO_2_ (%)	48.2 ± 23.1	33.9±19.2	42.8±10.2	34.9±16.5	0.5
pH	7.27±0.04	7.31±0.17	7.37±0.05	7.35±0.10	0.4
PaCO_2_ (mm Hg)	59.0±7.8	53.5±20.9	49.5±5.6	52.3±8.2	0.3
PaO_2_ (mm Hg)	36.7±17.7	24.0±8.1	27.0±4.3	28.3±8.8	0.3

Characteristics of the Cont, Sham, INJ and INJ+UCO lambs groups in this study and their fetal blood gases at the time of intubation and cord clamping. Data are presented as mean ± SD.

### Structural MRI

There was no overt structural injury evident on T1, T2, or SWI images in any group. However, MRS and DTI showed subtle brain injury, as detailed below.

### Magnetic resonance spectroscopy

**Fig 2:**
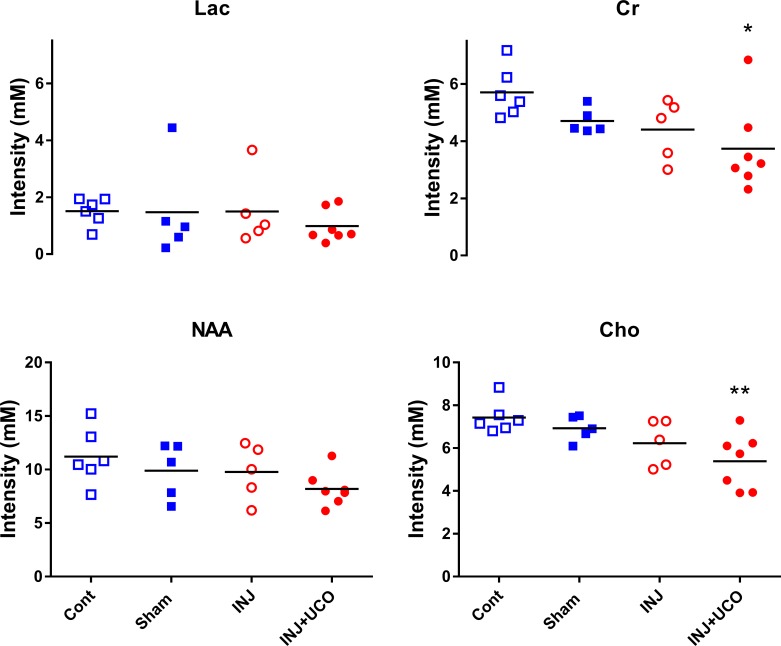
Absolute magnetic resonance spectroscopy (MRS) concentrations of metabolites in the preterm lamb brain. Individual absolute concentrations of lactate (Lac), creatine (Cr), choline (Cho), and N-acetylaspartate (NAA) using a single-voxel MRS encompassing supratentorial central white matter and deep grey matter in the unoperated control (Cont; blue open squares n = 6), sham surgery (Sham; blue closed squares n = 5), injurious ventilation (INJ; red open circles n = 5), and INJ + umbilical cord occlusion (INJ+UCO; red closed circles n = 7) groups. One way ANOVA comparisons tests relative to the Cont group were used with *P*<0.05 was considered statistically significant. *P<0.01, **P<0.001 vs INJ+UCO.

The MRS peak-area ratios of Lac to other metabolites, Cr, NAA, and Cho at 24 h were not different between groups (S3 Fig). However, absolute concentrations of Cr and Cho were significantly lower in the INJ+UCO group than the Cont group (*P* = 0.03, *P* = 0.009, respectively); no other differences were detected between the groups. Absolute concentrations of Lac and NAA at 24 h were not different between groups (Fig 2).

### Region of interest analysis for diffusion tensor imaging

**Fig 3:**
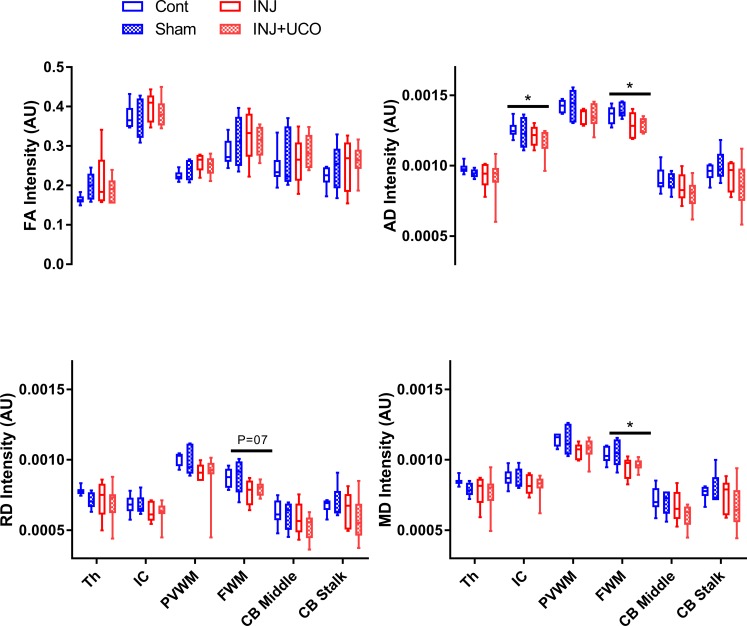
Mean and confidence intervals of DTI measurements for the small regions of interest analyses. Mean fractional anisotropy (FA), axial diffusivity (AD), radial diffusivity (RD), and mean diffusivity (MD) measurements for each region of interest (ROI) in the unoperated control (Cont n = 6), sham surgery (Sham n = 5), injurious ventilation (INJ n = 5), and INJ + umbilical cord occlusion (INJ+UCO n = 7) groups. The ROIs shown in S1 Fig were located in the thalamus (Th), internal capsule (IC), periventricular white matter (PVWM), frontal white matter (FWM), middle cerebellum (CB), and CB stalk. *P<0.01, **P<0.001 vs INJ+UCO.

DTI-ROI analysis for each area conducted in six Cont, five Sham, five INJ, and seven INJ+UCO lambs. AD was significantly decreased in the IC and FWM in INJ and INJ+UCO compared to Cont lambs (*P* = 0.05, *P* = 0.04, respectively), as shown in Fig 3. A trend was evident for lower RD in the FWM of INJ and INJ+UCO lambs compared to Cont lambs (*P* = 0.07). MD significantly decreased in the FWM of the INJ and INJ+UCO compared to the Cont group (*P* = 0.04), while mean AD, RD, and MD indices were lower in some INJ and INJ+UCO lambs compared to the Cont group (Fig 3). There were no mean differences in the Th, PVWM, middle CB, or CB stalk between groups. However, INJ+UCO, INJ, and Sham lambs showed significantly increased heterogeneity of diffusivity in the PVWM and CB stalk regions (S4 Fig).

### Whole brain intensity analysis

Whole-brain intensity distribution analysis was employed to describe the tensor orientation in the WM tract direction. This technique revealed lower AD, RD, and MD values that were closely occurred with the specific WM regions in this study (Table 2).

**Table 2:** Percentage of lambs found to have AD, RD, and MD in the selected WM regions of each group below the colour map threshold intensity 90% confidence interval of the Cont group.

	Cont *n* = 6			Sham *n* = 5			INJ *n* = 5			INJ+UCO *n* = 7		
	AD	RD	MD	AD	RD	MD	AD	RD	MD	AD	RD	MD
Th	0	0	0	20	80	60	20	80	60	43	86	86
IC	0	0	0	0	80	80	20	60	60	29	86	86
PVWM	0	0	0	0	20	0	20	80	40	14.3	86	43
FWM	0	0	0	0	20	20	40	80	60	43	86	71.4
CB	16.6	16.6	16.6	40	40	40	80	80	60	86	86	43

Thalamus (Th), internal capsule (IC), white matter (WM), periventricular WM (PVWM), frontal WM (FWM), and cerebellum (CB) vermis. DTI parameters: axial diffusivity (AD), radial diffusivity (RD), and mean diffusivity (MD). The DTI colour-mapping technique revealed the number of lambs found to have lower diffusivities from the total number (*n*) in each group. Therefore, there were different degrees of red colour (threshold range) between the lamb groups. Data are given as percentage of each group.

**Fig 4:**
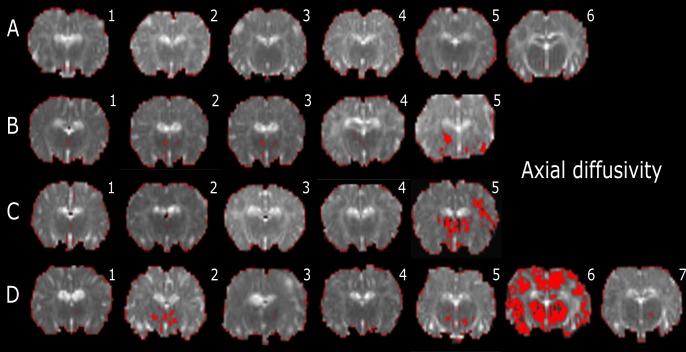
Representative slices of whole-brain DTI colour maps of the thalamus (Th) for axial diffusivity (AD). Voxel diffusivity intensities falling below the low threshold are shown as red for AD measurements for all lamb brains. All low-diffusion maps are overlaid on diffusion images for a slice passing through the thalamus (Th). In the injurious ventilation (INJ n = 5; C) and umbilical cord occlusion (INJ+UCO n = 7; D) groups, red indicates lower values in the range of the threshold, while black indicates values below the threshold (see explanation in S2 Fig). One sham surgery (Sham n = 5; B), one INJ, and three INJ+UCO lambs had widespread lower diffusivity values in the Th than unoperated control lambs (Cont n = 6; A).

**Fig 5:**
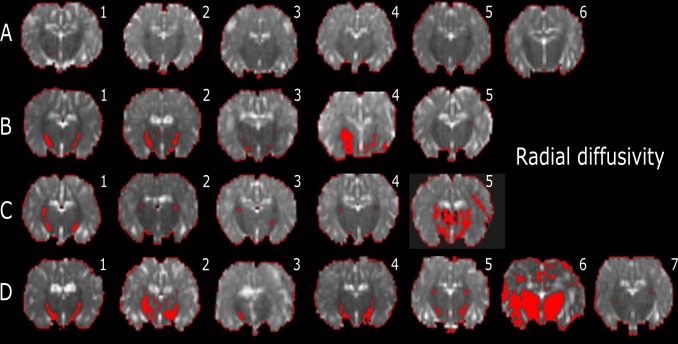
Representative slices of whole-brain DTI colour maps of the thalamus (Th) for radial diffusivity (RD). Voxel diffusivity intensities falling below the low threshold are shown in red for RD measurements for all lamb brains. All low-diffusion maps are overlaid on diffusion images for a slice passing through the Th. All lambs in the unoperated control (Cont n = 6; A) group had no low RD values in the Th, but low RD values appeared in the Th of 4 lambs following sham surgery (Sham n = 5; B), 4 lambs following injurious ventilation (INJ n = 5; C), and 6 lambs following umbilical cord occlusion (INJ+UCO n = 7; D).

**Fig 6:**
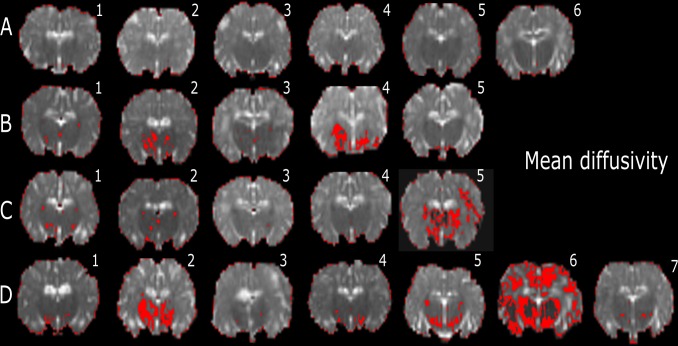
Representative slices of whole-brain DTI colour maps of the thalamus (Th) for mean diffusivity (MD). Voxel diffusivity intensities falling below the low threshold are shown in red for MD measurements for all lamb brains. All low-diffusion maps are overlaid on diffusion images for a slice passing through the Th. All lambs in the unoperated control (Cont n = 6; A) group had no low MD values in the Th, but low MD values appeared in the Th of about lambs following sham surgery (Sham n = 5; B), lambs following injurious ventilation (INJ n = 5; C), and lambs following umbilical cord occlusion (INJ+UCO n = 7; D).

**Fig 7:**
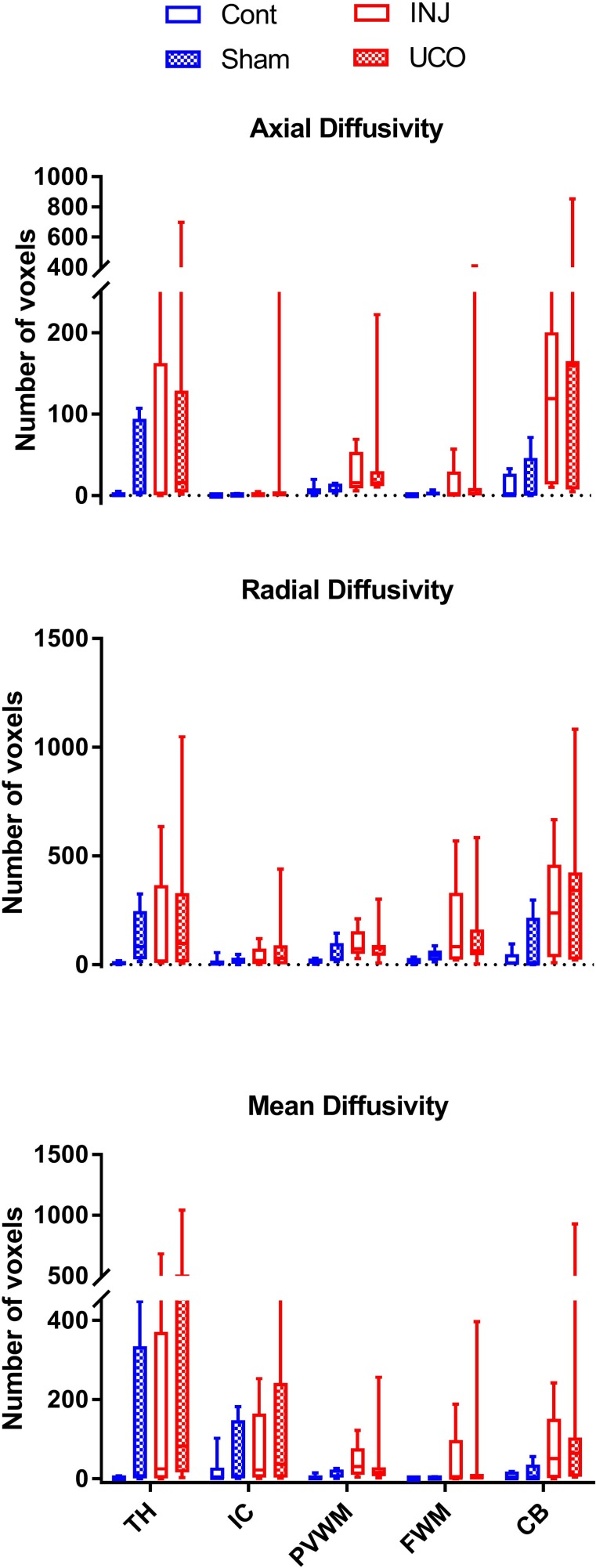
Comparison of voxel-based colour-map images for the whole brain. Images were compared for axial diffusivity (AD), radial diffusivity (RD), and mean diffusivity (MD) values below the threshold for specified regions in the lamb brain in all groups. The white matter (WM) regions were located in the thalamus (Th), internal capsule (IC), periventricular WM (PVWM), frontal WM (FWM), and cerebellum (CB) vermis. There were widespread lower AD, RD, and MD intensities in all WM regions in the umbilical cord occlusion (INJ+UCO n = 7; red closed circles) and injurious ventilation (INJ n = 5; red open circles) groups, but this did not reach statistical significance. While there were few lower diffusivities in WM regions in the sham surgery (Sham n = 5; blue closed squares) group the unoperated control (Cont n = 6; blue open squares) lambs showed very few voxels of low AD, RD, and MD intensities.

AD, RD, and MD histogram distributions for the whole brains of individual lambs are plotted in S2 Fig. In the low-diffusivity maps, there were different degrees of red colour (threshold range) within each specific brain region. We observed that the red shading was more visible in the Th of AD, RD and MD maps (Figs 4–6) in the INJ+UCO and INJ lambs, while the lowest diffusivity values were almost absent in the same region in the Cont group. Further, lower diffusivity values were more widespread in the surrounding areas of the Th of the INJ+UCO group than the INJ group and Sham group (Figs 4–6). Likewise, DTI diffusivities were reduced in the IC, PVWM, FWM and CB of some INJ+UCO and INJ lambs, but rarely in the Cont group (Table 2). Finally, quantifying the low-diffusivity voxels in each ROI, we compared voxel counts between all groups (Fig 7). We found evidence of reduced diffusivity in all WM regions in the INJ+UCO and INJ groups compared with Cont lambs. Interestingly, more lambs in the INJ+UCO group had some evidence of reduced AD, RD and MD values compared to the lambs INJ lambs (Fig 7), but this did not reach statistical significance.

## Discussion

Brain injury remains a significant burden for preterm infants, resulting in chronic neurological pathology including cerebral palsy [19], disrupted brain microstructures, and chronic myelination disturbances [20]. We demonstrated previously that injurious ventilation of preterm neonates upon delivery causes cerebral inflammation and haemodynamic instability [12], resulting in early markers of WM injury, detectable within the first 1–2 h after delivery with clinical 3T MRI [13,14]. While the previous study demonstrated early mechanisms of brain injury so soon after birth, it is not known whether these early markers of ventilation induced brain injury manifest as gross injury 24 h later. This is important given that the earliest clinical MRIs are likely to be achieved is at this 24h time-point. Since VIBI occurs through two major pathways, we also aimed to determine the relative contribution of each pathway to brain injury. We found no differences in indices of brain injury between the groups using conventional MRI techniques, including T1 and SWI. However, DTI-ROI analysis provides evidence that both the INJ and INJ+UCO groups had increased injury compared to controls. Further, the analysis of the whole brain using colour mapping threshold approach revealed evidence of worse pathology in the INJ+UCO lambs compared to INJ alone.

The initiation of ventilation using an injurious strategy results in acute brain inflammation and injury through two major pathways: an inflammatory cascade and haemodynamic instability. In order to isolate the relative contribution of the two pathways, we initiated ventilation in preterm lambs with or without an intact umbilical cord. Ventilating whilst maintaining placental blood supply prevents rapid swings in pulmonary and carotid blood pressures and flows and stabilizes cardiac output upon transition at birth [15]. The resulting injury from this ventilation strategy can be attributed to the inflammatory pathway only. Occluding the cord simulates immediate cord clamping at ventilation onset [15], which initiates both the inflammatory and haemodynamic pathways of brain injury. Any additional injury above that of the INJ group can therefore be attributed to the haemodynamic pathway.

DTI can provide microstructural information about the integrity of axons and myelin in the preterm infant brain; along the fiber tract (i.e., AD), decreased water diffusion indicates axonal degeneration, while perpendicular to the fiber tract (i.e., RD), decreased water diffusion indicates myelin loss [21]. Similar to Bassi et al. [22], who previously observed FWM lesions in preterm infants scanned in the neonatal period, we found DTI-ROI were able to detect decreased AD, RD and MD in the FWM due to injurious ventilation, with and without cord occlusion. Further, we found evidence of decreased AD in the IC in the INJ and INJ+UCO groups, which may indicate axonal loss [23] and may be associated with motor dysfunction later in development [24]. Further, subsequent studies have confirmed that decreased AD was observed with dysmyelination and demyelination [25,26]. We did not observe any significant changes in the thalamus, PVWM or CB in any lambs groups.

In contrast to the DTI-based ROI approach, whole-brain analysis is more sensitive to detect subtle brain injury, particularly axonal and myelin injury [27]. In this study, INJ+UCO lambs had the lowest AD, RD, and MD values in the WM regions compared to the other groups (Table 2). This suggests that activation of the inflammatory and haemodynamic pathways of injury had an additive effect on WM injury [28,29]. Importantly, it also suggests that improving the haemodynamic transition at birth (delayed cord occlusion) only reduces a portion of VIBI, if ventilation is delivered in an injurious fashion [12]. Therefore, simply improving the haemodynamic transition at birth is not enough to reduce VIBI–future therapies need to target the inflammatory cascade resultant from ventilation.

*In-vivo* MRS provides non-invasive metabolic information about the newborn’s brain [30]. Cr, Cho, Lac and NAA are the most readily determined resonances (peaks) using MRS [30]. The putative roles of Cr, Cho, Lac and NAA have been reported in many MRS studies. For instance, Cr is often used as an internal reference, but since it has been shown to change with neurological conditions and disease, changes in total Cr must always be considered [31]. A ^1^H-MRS study in infants within the first 4 days after birth demonstrated that a reduction in absolute brain Cr was associated with both the normal/mild and severe/fatal outcome groups, and may reflect reduced overall cellularity in neonatal encephalopathy groups compared with controls [32]. Cho is frequently used for the investigation of normal cell function and as a marker for cell membrane metabolism [33,34], and hence reduced Cho reflects reduced cellularity [35], impairments with myelination or altered cell types [36]. NAA is considered as a marker of neuronal density and function [37], and it is diffused along the axons; however, it breaks down in oligodendrocytes. Thus reductions in NAA may reflect neuronal or axonal degeneration [31]. Lac is an indicator of anaerobic metabolism. The elevated levels of brain Lac are thought to be due to major cell death events resulting from tumours and ischaemic diseases [38,39]. For any biomarker, the sensitivity of the MRS recordings and the sample size acquired are important considerations as to whether absolute concentrations and/or the metabolic ratios are used for quantification of neural metabolic changes [40]. In this study, we found that INJ+UCO lambs had significantly reduced absolute Cr and Cho values compared to controls, but no significant differences in Lac or NAA, at 24 h. An early decline in absolute brain Cr in this study might reflect impending tissue loss, as suggested first in a study on shaken baby syndrome [41]. MRS recordings on babies with shaken baby syndrome showed a decline in Cr and NAA concentration and a delayed, but pronounced increase in Lac, indicative of tissue loss and reduced cellularity, which was able to be measured at a much earlier stage than with conventional MRI [41]. The INJ+UCO lambs had lower Cho levels than the control group. Others have previously associated low Cho levels in the brain with reduced myelination [42]. We have previously shown that alterations in DTI measurements at the time of preterm delivery correlated with impairment of neuron and myelination as a result of injurious mechanical ventilation [43]. Measuring myelination was outside the scope of this study, but the loss of oligodendrocyte progenitors has been well characterized in models of prematurity [44], HIE [45], and ventilation [7]. Lactate concentrations were not significantly different between the lamb groups at 24 h in this study. Others have also reported that there was no significant Lac elevation in preterm infant brains [46]. Furthermore, we found no significant differences in MRS peak-area metabolite ratios at 24 h, which might reflect the relative insensitivity of these indices in detecting the early onset of brain injury. Others also failed to detect an effect of magnesium sulfate, which was expected to decrease the severity of delayed cerebral energy failure in infants with cerebral ischaemia, on the MRS peak-area ratios of Lac to other metabolites within the first 1–2 days of postnatal life [47]. Our metabolite concentration findings greatly improve our understanding for pathological interpretation compared with that provided by peak-area ratios.

We did not observe obvious lesions that have been detected in animal models with conventional MRI at 24 h [48]. However, this does not rule out the possibility such lesions might manifest in the following days. Brown et al. [49] reported that there was a direct relationship between poor behavioural performance and severe microstructural WM abnormalities on MRI findings at 5–7 days of age, and at two years of age in preterm infants. An ultimate aim of our research is to identify a marker of preterm brain injury before it manifests to allow a window for treatment. Our previous study did not observe DTI changes using DTI-based ROI approach at one hour after delivery [14]. Identification of DTI-based ROI approach with subsequent structural injury in longer-termed studies will be critical to validate this technique as a reliable early marker of brain injury [50,51].

The DTI findings in this study confirm and extend our previous investigations of neuropathology in preterm neonate brains. However, we acknowledge that the study has important limitations. Histological examination of the lamb brains in this study was not undertaken to confirm inflammatory markers or subtle WM injury. However, we have previously demonstrated that alterations in fractional anisotropy and diffusivity at the time of preterm delivery correlated with impairment of myelination as a result of injurious mechanical ventilation [43]. This study and previous studies [14,43], show that MRI can be quantitative for microstructural changes in specific anatomical structures in the preterm brain, providing potentially useful biomarkers for preterm delivery–associated neuropathology in the clinic. A further limitation, is that the surgery in of itself is injurious, since there was mild injury observed in the sham lambs compared to the Cont group. This is likely due to using twins exposed to longer isoflurane which is harmful to the preterm brain [52].

In conclusion, both DTI and colour-map threshold technique revealed that VIBI is associated with very low AD, RD, and MD intensities observed in the WM of preterm lambs 24 h after ventilation. When both pathways of injury were initiated WM injury was amplified compared to the inflammatory pathway alone, demonstrating the importance for targeting of both pathways of injury for any therapeutic strategy focused on reducing VIBI.

## Supporting information

S1 Fig Magnetic resonance imaging (MRI) data analysis regions of interest (ROIs). Examples of ROIs in specific regions of (A) the thalamus (Th), (B) internal capsule (IC), (C) periventricular white matter (PVWM), (D) frontal white matter (FWM), and the cerebellum (CB) vermis, targeting (E) midline and (F) stalk white matter for DTI analysis. Figure is reproduced from Alahmari et al. [14].
(TIF)

S2 Fig DTI histogram distributions for individual lambs. Histogram distribution plots of axial diffusivity (AD), radial diffusivity (RD), and mean diffusivity (MD) for the whole brains from each group. Blue lines represent the unoperated control (Cont) group; blue dashed lines represent the sham surgery (Sham) group. Red lines represent the injurious ventilation (INJ) group, while red dashed lines represent the lambs exposed to umbilical cord occlusion (INJ+UCO) during the initial 15 min of high tidal-volume (V_T_) ventilation. The black box represents the 75% confidence interval (CI) of all voxel intensities observed in the distribution in Cont lamb brains. Threshold (green dashed line) applied to the mapped intensities in the overlay in all groups.
(TIF)

S3 Fig Peak-area magnetic resonance spectroscopy (MRS) lactate metabolite ratios. Individual MRS peak-area metabolite ratios utilising a single-voxel encompassing supratentorial central white matter and deep grey matter in the unoperated control (Cont; blue open squares), sham surgery (Sham; blue closed squares), injurious ventilation (INJ; red open circles), and injurious ventilation with umbilical cord occlusion (INJ+UCO; red closed circles) groups. Lac: lactate; Cr: creatine; Cho: choline; NAA: N-acetylaspartate.
(TIF)

S4 Fig Standard deviation of DTI measurements. The standard deviation of the distribution of voxel intensities within each region of interest ROI for each animal was calculated for fractional anisotropy (FA), axial diffusivity (AD), radial diffusivity (RD), and mean diffusivity (MD) measurements in the unoperated control (Cont), sham surgery (Sham), injurious ventilation (INJ), and umbilical cord occlusion (INJ+UCO) groups. The means of the standard deviations were tested for differences between the four groups of lambs using one-way ANOVA. The ROIs shown in S1 Fig were located in the thalamus (Th), internal capsule (IC), periventricular white matter (PVWM), frontal white matter (FWM), cerebellum (CB) middle and cerebellum (CB) stalk. * *P* < 0.01 and ** *P* < 0.001 group effect.
(TIF)
