# Supplementary figures and images for: Diffusion tensor imaging detects ventilation-induced brain injury in preterm lambs
Source: PLoS One. 2017 Dec 6;12(12):e0188737. doi: 10.1371/journal.pone.0188737 (PMC5718608; doi:10.1371/journal.pone.0188737)

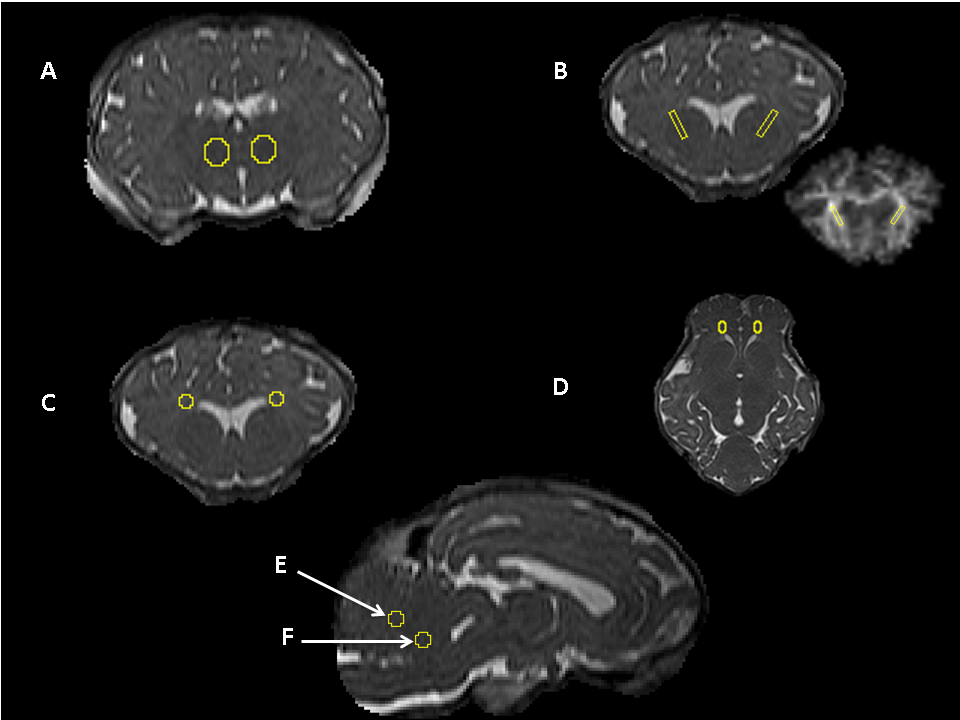

Supplement: S1 Fig — Examples of ROIs in specific regions of (A) the thalamus (Th), (B) internal capsule (IC), (C) periventricular white matter (PVWM), (D) frontal white matter (FWM), and the cerebellum (CB) vermis, targeting (E) midline and (F) stalk white matter for DTI analysis. Figure is reproduced from Alahmari et al. [14]. (TIF) [file pone.0188737.s001.tif]

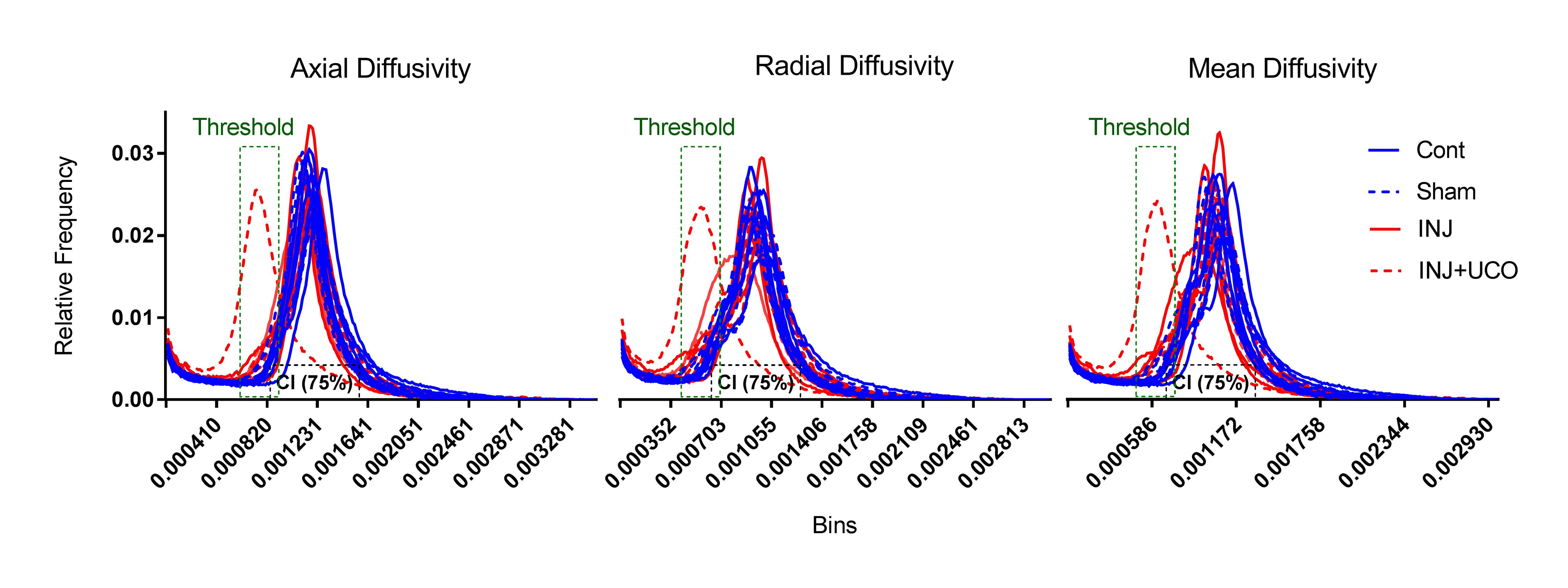

Supplement: S2 Fig — Histogram distribution plots of axial diffusivity (AD), radial diffusivity (RD), and mean diffusivity (MD) for the whole brains from each group. Blue lines represent the unoperated control (Cont) group; blue dashed lines represent the sham surgery (Sham) group. Red lines represent the injurious ventilation (INJ) group, while red dashed lines represent the lambs exposed to umbilical cord occlusion (INJ+UCO) during the initial 15 min of high tidal-volume (VT) ventilation. The black box represents the 75% confidence interval (CI) of all voxel intensities observed in the distribution in Cont lamb brains. Threshold (green dashed line) applied to the mapped intensities in the overlay in all groups. (TIF) [file pone.0188737.s002.tif]
